# Long‐Term Results of UPPP and Coblation Channeling of the Tongue for Obstructive Sleep Apnea

**DOI:** 10.1002/wjo2.70058

**Published:** 2025-10-13

**Authors:** Ren‐Hui Chen, Qian Cai, Wei‐Qi Chen, Xiao‐Ming Huang

**Affiliations:** ^1^ Department of Otolaryngology, Head Neck Surgery, Sun Yat‐sen Memorial Hospital Sun Yat‐sen University Guangzhou Guangdong China

**Keywords:** obstructive sleep apnea, tongue radiofrequency, uvulopalatopharyngoplasty

## Abstract

**Objective:**

To evaluate the long‐term surgical outcomes of combined uvulopalatopharyngoplasty (UPPP) and coblation channeling of the tongue (CCT) for moderate‐to‐severe obstructive sleep apnea (OSA).

**Methods:**

The study enrolled patients with moderate‐to‐severe OSA who underwent treatment with UPPP plus CCT and had a minimal follow‐up of 2 years. Postoperative clinical assessment including apnea‐hypopnea index (AHI) and lowest oxygen saturation (LSAT) in Polysomnography (PSG), Epworth Sleep Scale (ESS) values in sleep questionnaires, and cephalometric variables were compared with the preoperative baseline data.

**Results:**

Thirty‐six patients with complete records and follow‐up were enrolled. According to the data of minimal 2 years postoperative, AHI had significantly decreased from 39.6 ± 19.8 to 18.2 ± 19 events/h (*p* < 0.0001), and ESS had remarkably improved from 9.6 ± 5.6 to 6.2 ± 4.8 (*p* < 0.0001). Morbidity of the surgery was low, and no long‐term complications occurred. Superior movement of the hyoid bone was observed on postoperative cephalometry.

**Conclusion:**

UPPP plus CCT provides a safe and long‐term effective treatment for moderate‐to‐severe OSA with the advantages of minimal invasive and single‐stage intervention.

## 1 Introduction

Upper airway obstruction during sleep is the hallmark of obstructive sleep apnea (OSA). While continuous positive airway pressure (CPAP) remains the first‐line therapy for moderate‐to‐severe OSA, poor adherence (29%–83%) limits its effectiveness [1]. Surgical interventions remain a beneficial alternative for CPAP‐intolerant patients.

Uvulopalatopharyngoplasty (UPPP) is the most common surgical approach for velopharyngeal obstruction, yet its standalone surgical rate in multilevel OSA is 41% [2], and the average residual apnea–hypopnea Index (AHI) is 29.8 events/h [3]. Drug‐induced sleep endoscopy (DISE) studies reveal that 68.2% of OSA patients exhibit multilevel collapse [4], underscoring the need for combined interventions targeting both retropalatal and retroglossal regions.

Coblation channeling of the tongue (CCT), a minimally invasive radiofrequency ablation, addresses tongue base hypertrophy. Short‐term studies report a 35.5%–66.7% success rate for UPPP combined with CCT [5], but long‐term outcomes remain poorly characterized. Furthermore, surgical success (defined as a reduction in AHI > 50% and < 20 events/h) has not been rigorously validated beyond 6 months [2]. This study aimed at assessing 2‐year outcomes of modified UPPP plus CCT for moderate‐to‐severe OSA, while identifying predictors of surgical failure.

## 2 Methods

### 2.1 Participants

This study enrolled patients who were treated with modified UPPP plus CCT at our department between July 2019 and May 2022. Dr. Chen R‐H was the surgeon and DISE examiner. All patients were diagnosed with moderate to severe OSA (AHI > 15 events/h) based on physical examination, fiberoptic laryngoscopy, X‐ray lateral cephalometry, and polysomnography (PSG) findings as outpatients. Patients who refused or did not adhere to CPAP were recommended for surgery. Inclusion criteria were: age, 18–65 years; body mass index (BMI) < 35 kg/m^2^; Friedman tongue position (FTP), 3–4 or FTP 2 with cephalometry demonstrating minimal posterior airway space (PAS) < 10.9 mm or mandibular plane to hyoid bone distance (HMP) > 15 mm [6, 7]; and complete baseline records (including X‐ray cephalometry, and PSG). Exclusion criteria were severe craniofacial deformities, obstruction in the nasal and nasopharyngeal areas, severe cardiovascular and cerebrovascular diseases and respiratory failure or American Society of Anesthesiologists classification grade > 3 and previous airway surgery for OSA. Ethical approval was obtained from the ethics committee of our hospital. Written informed consent was obtained from all participants.

### 2.2 Surgical Treatment

The patient was administered with general anesthesia via orotracheal intubation. The surgical procedure was modified to include UPPP plus CCT. Modified UPPP mainly avoids aggressive dissection in the middle of the soft palate and uvula and enlarges the retropalatal space by laterally fixating the posterior pillar and palatopharyngeal muscle. CCT was performed using the 22‐G radiofrequency needle electrode (EIC4855‐01, Smith & Nephew ENT, Andover, MA, USA) at a power setting of 6 for 15 s per port according to the surgical techniques described by MacKay and colleagues [8]. For patients in Friedman tongue position 3, seven‐port channeling coblation, including three midline channels and two lateral channels on each side, was performed. For Friedman tongue position 4, one channel was added on each lateral side, and nine‐port channeling coblation was used for CCT.

All patients were extubated immediately after surgery and admitted to the general ward until discharge 2 days postoperatively. Glucocorticoids and cephalosporins were routinely administered intraoperatively for 2 days. The patient was discharged with oral antibiotics for 1 week. All patients received comprehensive lifestyle modifications guidance, including adoption of a low‐carbohydrate and Mediterranean diets, and regular aerobic exercise (30 min of walking or jogging 3–5 days per week) [9]. Further, nasal steroids were prescribed.

### 2.3 PSG

Baseline PSG was performed 1 week to 1 month preoperatively. Postoperative PSG 1 and 2 were obtained at 6 months and a minimum of 2 years after surgery, respectively. Overnight PSG data were collected at our sleep laboratory using an in‐laboratory PSG device (Alice 6 LDx INTL base station, Philips, USA) in priority. Six patients in this group, using a portable sleep apnea recording device (Alice PDx, Philips, USA) preoperatively, were collected the PSG data by the same recording device after surgical treatment. The in‐laboratory PSG recorded 16 channels, including electroencephalography, electrooculography, and electromyography of the chin and tibialis; oronasal thermistor and flowmetry; finger pulse oximetry; abdominal and thoracic respiratory movements; snoring; electrocardiography; and body position. From portable PSG, the electroencephalogram, electrooculogram, and electromyogram of the chin and tibialis were missing. The parameters were defined according to the principles of the American Academy of Sleep Medicine version 2014.

### 2.4 Outcome Measures

The primary outcomes used to compare surgical efficacy between baseline and postoperative 6 months and a minimum of 2 years were AHI, lowest oxygen saturation (LSAT), and Epworth Sleep Scale (ESS) values. The evaluation included physical examination, relief of OSA symptoms, and surgical morbidities. The surgery was considered effective (responder) at an AHI < 20 events/h with an AHI reduction > 50% at 2 years postoperatively compared to baseline, and cured when the resident AHI < 5 events/h [2].

Symptom relief was recorded according to patients' self‐reported OSA symptoms and compared to the preoperative baseline status. Symptoms included dry throat, globus sensation, suffocation, headache, fatigue, and snoring. The bed partner's visual analog scale (VAS) evaluation of snoring (0–10) was used for snoring assessment [10].

Comparison of upper airway morphology between baseline and postoperative was visualized with lateral cephalometry in the upright position. The included cephalometric parameters were referred to the published studies [11, 12, 13]. Nonlinear variables such as the area of tough was measured by image J. The measurement of the cephalometry was taken by sleep professional Chen W‐Q who was blinded to the surgical outcomes.

### 2.5 Statistical Analysis

Statistical analyses were performed using the Python. The normality of the data distribution was confirmed using the Kolmogorov–Smirnov test. Continuous variables are expressed as mean ± standard deviation. The paired Student's *t*‐test and ANOVA with Dunnett's multiple comparisons test were used to compare the change between baseline and postoperative mean values. The ratio of means was used to assess the surgical efficacy. Fisher's chi‐square test was used to assess the differences between responders and non‐responders. Binary logistic regression analysis was used to identify the predictors of surgical responders. Statistical significance was set at a *p*‐value < 0.05.

## 3 Results

### 3.1 Baseline Characteristics

We had 47 patients with moderate‐to‐severe OSAHS who had been treated with the modified UPPP plus CCT during July 2019 and May 2022. Eleven patients (23.4%) were lost to follow‐up or refused to undergo PSG when 2 years after surgery. Eventually, 36 patients entered this study and completed PSG at baseline and PSG 1 and PSG 2 at postoperative follow‐ups. All 36 participants were men, with a mean age of 36.7 ± 9.4 (range 24–58) years. The median follow‐up was 33.5 (range 24–54) months. The baseline characteristics of the cohort are shown in Table 1, the distribution of obstruction in DISE examination using the VOTE classification system are illustrated in Table 2.

**Table 1:** General information and baseline clinical variables in patients with OSA who underwent uvulopalatopharyngoplasty plus coblation channeling of the tongue.

Variables	Values (*n* = 36)
Male gender (*n*, %)	36, 100%
Age (mean ± SD, years)	36.7 ± 9.4
BMI (mean ± SD, kg/m^2^)	26.7 ± 3.3
AHI (mean ± SD,/h)	39.6 ± 19.8
LSAT (mean ± SD, %)	71 ± 16.5
Self‐evaluated ESS score (mean ± SD)	9.6 ± 5.6
Friedman Stage I, II, III [*n* (%)]	2 (5.6), 14 (38.9), 20 (55.5)
Friedman tonsillar Grade 1, 2, 3, 4 [*n* (%)]	6 (16.7), 23 (63.9), 5 (13.9), 2 (5.5)
Friedman tongue position Grade I, II, III, IV [*n* (%)]	0 (0), 11 (22.2), 23 (63.9), 2 (5.5)
Receded mandible [*n* (%)]	23 (63.9)
Inferiorly positioned hyoid bone [*n* (%)]	19 (52.8)
Narrowing retroglossal airway [*n* (%)]	11 (30.6)

Abbreviations: AHI, apnea‐hypopnea index; BMI, body mass index; ESS, Epworth Sleep Scale; LSAT, lowest oxygen saturation.

**Table 2:** Distribution of obstruction using the VOTE classification (*n* = 29).

	Direction [*n* (%)]					
	Anteroposterior		Lateral		Concentric	
Level	Partial	Complete	Partial	Complete	Partial	Complete
Velum	5 (11.2)	12 (41.4)	1 (3.4)	6 (20.7)	0 (0)	5 (11.2)
Oropharynx	—	—	4 (17.8)	14 (48.3)	—	—
Tongue base	15 (51.7)	10 (34.5)	—	—	—	—
Epiglottis	4 (17.8)	0 (0)	1 (3.4)	0 (0)	—	—

*Note:* —, no data.

### 3.2 Therapeutic Effects

**Figure 1:**
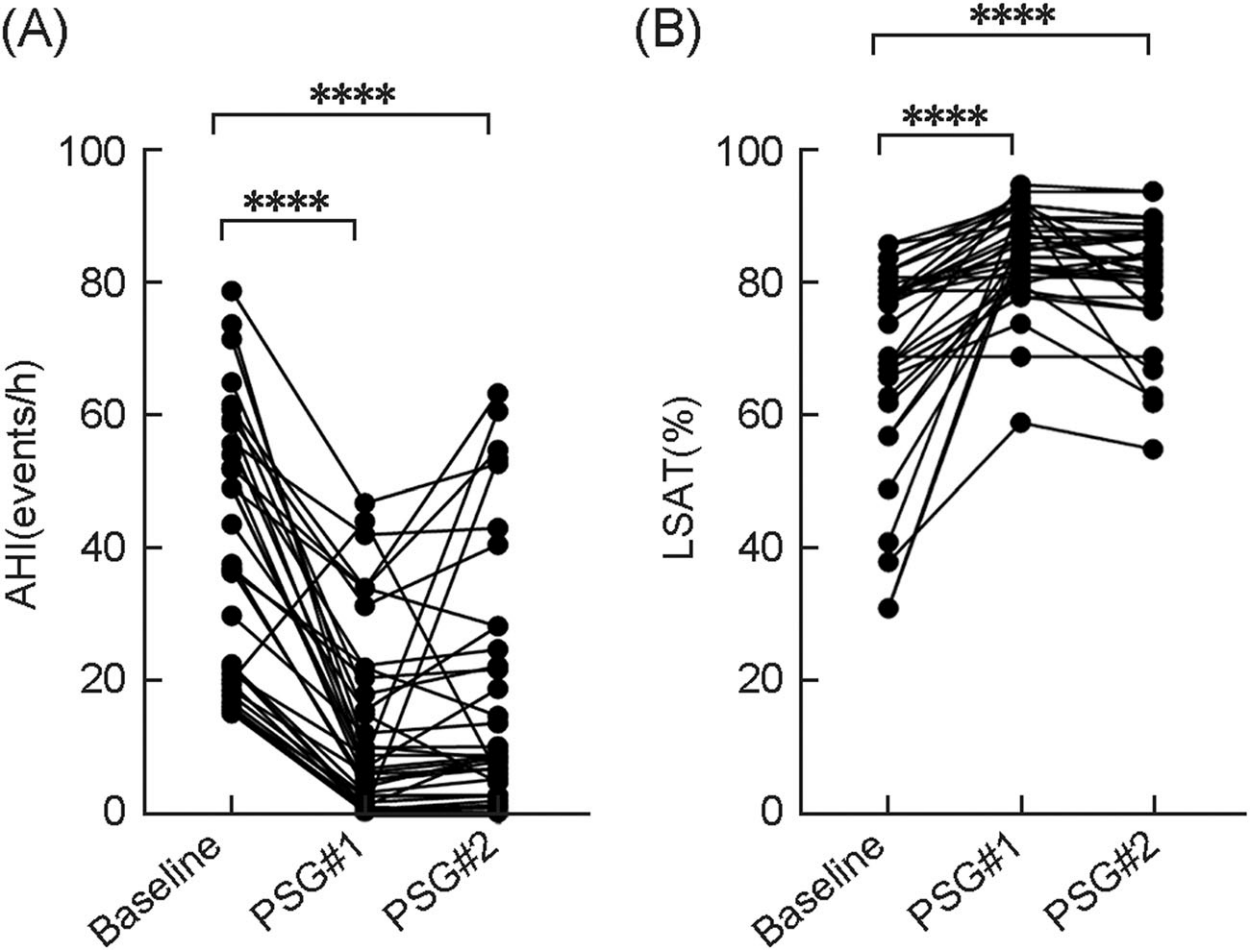
Lines showing the change of polysomnographic parameters. (A) The apnea‐hypopnea index (AHI) and (B) the lowest oxygen saturation (LSAT) on baseline and two postoperative polysomnography (PSG 1 at 6 months and PSG 2 at 2 years). ****p < 0.0001.

As shown in Table 3 and Figure 1, at 2‐year follow‐up, the overall mean AHI decreased by 53.4% [95% confidence interval (CI): 29.3%–69.5%, 39.6 ± 19.8 to 18.2 ± 19 events/h, *p* < 0.0001], with 66.7% (24/36) achieving surgical success (AHI < 20 events/h, with an AHI reduction > 50%). Notably, 22.2% (8/38) achieved complete remission (the resident AHI < 5 events/h). The LSAT improved from 71% to 81.4% (*p* < 0.0001), and ESS score decreased by 28% (95% CI: 13.1%–46.6%, 9.6 ± 5.5 to 6.2 ± 4.8, *p* < 0.0001). This suggests that the long‐term efficacy of UPPP plus CCT is favorable.

**Table 3:** The change of body mass index, polysomnographic parameters and Epworth sleep scale between baseline and postoperation.

Items				*p* value	
	Baseline	PSG 1	PSG 2	PSG 1 versus baseline	PSG 2 versus baseline
BMI (kg/m^2^)	26.7 ± 3.3	26.4 ± 3.2	26.5 ± 3	0.942	0.993
AHI (/h)	39.6 ± 19.8	13.5 ± 13.9	18.2 ± 19	< 0.0001	< 0.0001
LAST (%)	71 ± 16.5	84.6 ± 7.3	81.4 ± 10.4	< 0.0001	< 0.0001
ESS	9.6 ± 5.6	5.7 ± 4.4	6.2 ± 4.8	< 0.0001	< 0.0001

Abbreviations: AHI, apnea–hypopnea index; BMI, body mass index; ESS, Epworth Sleep Scale; LSAT, lowest oxygen saturation. [Correction added on 22 April 2026, after first online publication: 9.6.8 ± 5.6 has been corrected to 9.6 ± 5.6 in Table 3.]

### 3.3 Factors Predicting Effective Outcome

Of the 36 patients, 12 were non‐responders at the 2‐year follow‐up. A comparison of baseline parameters between surgical responders and non‐responders revealed that non‐responders exhibited a significantly higher AHI (50.2 ± 16.2 events/h vs. 34.1 ± 19.9 events/h, *p* = 0.021), with no other significant differences observed between the two groups (Table 4). The correlation analysis revealed that the baseline AHI (*r* = −0.383, *p* = 0.021), the tongue position *(r* = −0.323, *p* = 0.054), and Friedman stage (*r* = −0.294, *p* = 0.082) were associated with surgical response. Additionally, BMI was correlated with the baseline LSAT (*r* = −0.621, *p* < 0.01), and the Friedman tonsil stage was correlated with the baseline AHI (*r* = 0.654, *p* < 0.01). These findings suggest that individuals with higher BMI are likely to experience lower LSAT levels, and larger tonsils appear to be a determining factor for higher AHI values within our cohort.

**Table 4:** Comparison of baseline parameters between surgical responders and non‐responders.

Variables	Responders (*n* = 24)	Non‐responders (*n* = 12)	Statistics value	*p* value
Age (mean ± SD, years)	35.5 ± 9.8	39.2 ± 8.4	1.123	0.269
BMI (mean ± SD, kg/m^2^)	26.8 ± 3.7	26.3 ± 2.4	−0.397	0.694
AHI (mean ± SD,/h)	34.1 ± 19.9	50.2 ± 16.2	2.42	0.021
LSAT (mean ± SD, %)	72.5 ± 17.1	68.1 ± 15.4	−0.753	0.456
Self‐evaluated ESS score (mean ± SD)	9.7 ± 5.5	9.4 ± 6	−0.125	0.901
Friedman Stage [I/II/III, *n*]	2/11/11	0/3/9	3.118	0.21
Friedman tonsillar grade [1/2/3/4, *n*]	5/14/3/2	1/9/2/0	2.198	0.532
Friedman tongue position grade [II/III‐IV, *n*]	10/14	1/12	2.047	0.041
Receded mandible [Y/N, *n*]	15/9	9/3	0.563	0.453
Inferiorly positioned hyoid bone [Y/N, *n*]	13/11	6/6	0.056	0.813
Narrowing retroglossal airway [Y/N, *n*]	8/16	3/9	0.262	0.609

Abbreviations: AHI, apnea‐hypopnea index; BMI, body mass index; ESS, Epworth Sleep Scale; LSAT, lowest oxygen saturation.

**Figure 2:**
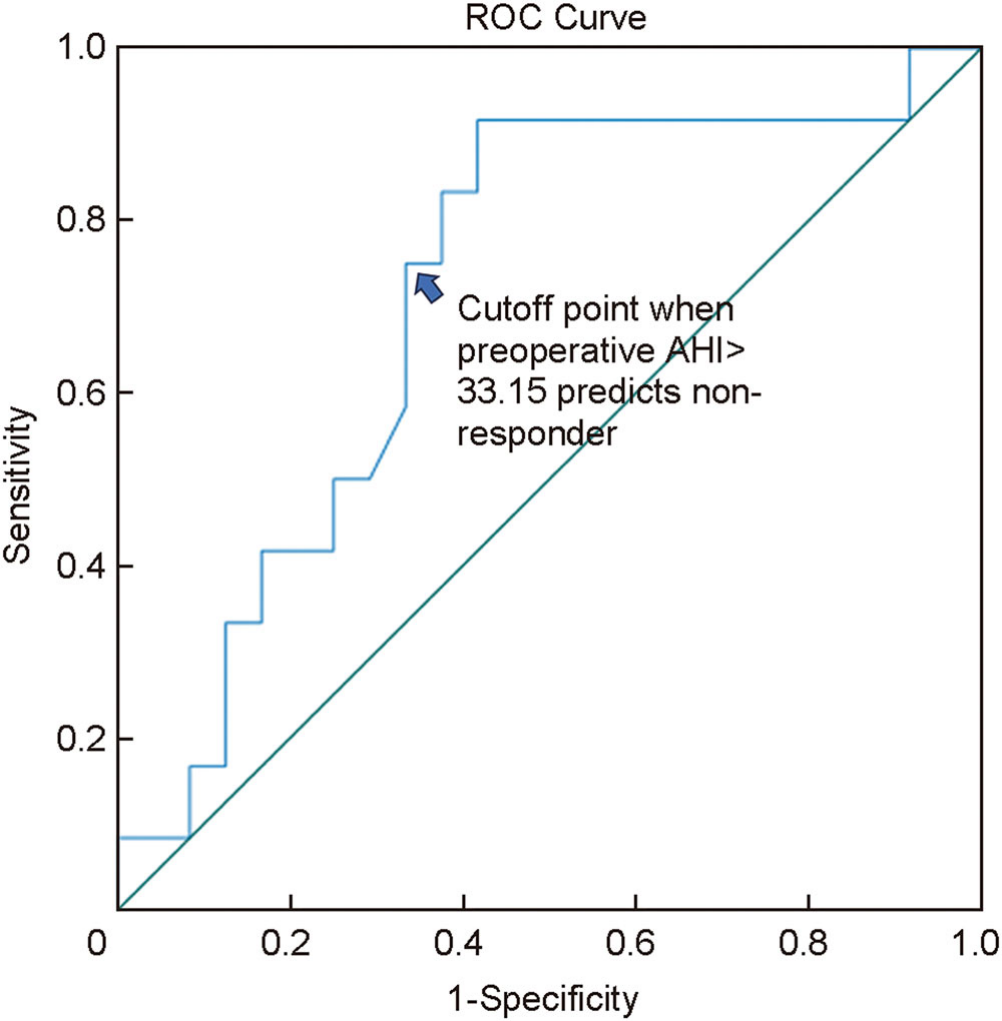
Receiver operating characteristics (ROC) curve for preoperative apnea‐hypopnea index (AHI) predicting surgical responders.

The variables significantly correlated with surgical response, including baseline AHI, tongue position, and Friedman stage, were analyzed using binary logistic regression analysis to identify the factors that determine surgical prognosis. The baseline AHI *(p* = 0.029) was identified as the independent factor for predicting surgical response (Table 5). ROC analysis was employed to determine the cutoff value of baseline AHI, which would predict the likelihood of benefiting from UPPP plus CCT. For surgical responders, area under the curve (AUC) was 0.714 (*p* = 0.039), and the optimal cutoff value for baseline AHI was 33.15 events per hour (Figure 2).

**Table 5:** Logistic analysis for the factors influencing surgical responders.

	Pearson's correlation		Binary logistic analysis		
Variables	*R* value	*p* value	*p* value	Odd ratio	95% CI
Baseline AHI	−0.383	0.021	0.029	0.943	0.895–0.994
Tongue position	−0.323	0.054	0.545		
Friedman stage	−0.294	0.082	0.127		

Abbreviations: AHI, apnea‐hypopnea index; CI, confidence interval.

### 3.4 Complications and Symptom Relief

Three cases of intraoperative bleeding from the tongue channeling port occurred and resolved with suturing. No major postoperative complications, such as airway obstruction, tongue infection, and velopharyngeal insufficiency occurred. Minor postoperative complications, mainly temporary dysphagia caused by pharyngeal pain, resolved significantly by postoperative day 2.

At the final follow‐up 2 years postoperatively, 4/36 patients still reported throat dry, 4/36 reported globus sensation, 8/36 had shortness of breath, 3/36 had headache, and 4/36 suffered with fatigue. The snoring level deteriorated in four patients and remained unchanged in six patients. Twelve (33.3%) patients reported moderate to severe self‐rated snoring score, VAS > 3 and affecting the sleep of bedmates.

### 3.5 Cephalometric Evaluation

**Figure 3:**
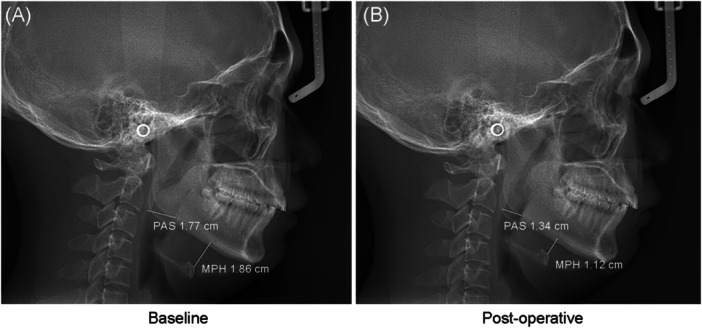
Preoperative and postoperative cephalograms of one typical case of OSA underwent uvulopalatopharyngoplasty plus coblation channeling of the tongue. (A) The baseline preoperative cephalogram. (B) The postoperative cephalogram. The distance between the mandibular plane and the hyoid bone (MPH) and posterior air space (PAS) was indicated with yellow lines.

The study had 12 patients with postoperative lateral cephalometries before and at least 6 months after treatment. Of these 12 patients, three were nonresponders. As shown in Figure 3, postoperative cephalometry indicated superior hyoid bone movement was observed postoperatively based on the shortened distances of mandibular plane to hyoid (MP‐H, 18.4 ± 5.3 mm vs. 15.0 ± 4.9 mm, *p* = 0.061). The distance of post airway space (PAS), the length and height and area of the tongue were not significantly decreased compared to baseline. The cephalometric evaluation raised that UPPP plus CCT effectively led to the superior movement of the hyoid bone.

## 4 Discussion

This study demonstrates sustained efficacy of UPPP plus CCT at 2 years, aligning with the Sleep Apnea Multi‐level Surgery (SAMS) randomized clinical trial [14], by surpassing earlier meta‐analyses reporting 35.5%‐66.7% success rates [5]. The superior outcomes may stem from stringent patient selection. In our series, we enrolled patients with tongue base hypertrophy not only according to the Friedman stage in tongue position but also with cephalometric evidence of inferior displacement of the hyoid bone and narrowing of the retroglossal pharyngeal space. Eleven of 36 (30.5%) of FTP 2 patients with hyoid inferior displacement were included, a subgroup previously underrepresented. Ten patients with FTP 2 were responded after and showed a trend of a superior response than those with FTP 3–4 (10/11 vs. 14/25, *p* = 0.041), indicating that a higher FTP may predict an increased risk of nonresponse to CCT.

A systematic review demonstrated a higher response rate to CCT combined with UPPP than to CCT alone. The advantages of CCT alone include the convenience of repeated operations under local anesthesia and its suitability for outpatient or office‐based clinics. The mean number of CCT treatment sessions was 3.4–4.5. The response rate for CCT alone can reach 33%–38% [3]. In the present series, we only used one session of CCT. For the 1/3 non‐responders, one more CCT treatment may be administered based on previous studies [15, 16].

In the present study, UPPP was the mainstay of treatment for OSA, even in patients with Friedman tonsil Stage 1–2. Considering that tonsil volume is not small in Friedman tonsil Stage 1–2, tonsil ablation is beneficial for reducing the velopharyngeal volume and enlarging the pharyngeal cavity laterally. The Muller maneuver is useful for excluding patients who adhere to UPPP [17]. Combining UPPP may be the major reason for the long‐term surgical success in the present series. The 66.7% success rate of surgery was consistent with the Australian multicenter clinical trial at 6‐months follow‐up [8] and superior than others [5], likely due to differences in surgical technique (e.g., lateral pharyngeal suspension in modified UPPP) and cohort characteristics (lower mean BMI: 26.7 kg/m^2^ vs. > 30 kg/m^2^ in western cohorts). ^8,17^Persistent snoring in 33.3% of patients highlights the procedure's limitations, warranting further DISE‐guided interventions or surgical approaches (e.g., maxillomandibular advancement, or upper airway stimulation).

Regarding the surgical responders, multivariate analysis revealed that the baseline AHI was the only independent predictor of long‐term effectiveness. The procedure of UPPP and CCT is most likely to be effective when baseline AHI is less than 33.15 events/h in our series. Further, Non‐responders positively correlated with the Friedman stage of OSA, and 9/12 non‐responders had Friedman stage 3 within our series, although the discrepancy (*p* = 0.082) did not reach statistical significance, suggesting that combined UPPP and CCT surgery is unfavorable for moderate to severe OSA with small tonsils and a hypertrophic tongue. Friedman et al reported a success rate of 43.8% of UPPP plus tongue‐base reduction using a radiofrequency technique (TBRF) for stage III patients [18], and the rate was 55% in our series. In addition, during the preliminary 18 cases of our practice, DISE suggests poorer surgical outcomes in four of five patients with circumferential velopharyngeal collapse. This is consistent with Hsu et al.'s study [19] in which concentric collapse of the velum was associated with a higher residual AHI. Herein, we endeavored to avoid performing surgery for patients with concentric collapse of the velum.

Skeletal abnormalities of the oropharyngeal cavity are frequently observed in patients with OSA. Inferior displacement of the hyoid bone and retrodisplacement or hypoplasia of the mandible were the most common abnormalities observed in southern China [7]. Surgical success showed no association with these two abnormal skeletal alterations in the present study. We postulated that CCT led to narrowing compliance of the tongue and addressed retroglossal collapse, which may contribute to therapeutic efficacy. This possibility was partly confirmed by cephalometric evaluation, which showed superior repositioning of the hyoid bone after CCT in this study. Radiofrequency ablation surgery leads to a reduction in soft tissue volume through thermic lesions and subsequent scar contraction effects, which may be the primary reason for the anterosuperior displacement of the hyoid bone position [20].

Channeling temperature‐controlled radiofrequency tongue ablation allows maximal tissue ablation with minimal thermal injury (< 85°C), and leads to limited postoperative pain and low morbidity. The combination of modified UPPP and CCT complies with the minimally invasive, single‐stage, and multilevel surgery for OSA [21]. No significant surgical complications, such as dyspnea caused by tongue swelling or abscess, and fewer postoperative dry and globus sensations of the throat occurred during the long‐term follow‐up in the present series, confirming the safety and minimal invasiveness of UPPP plus CCT.

This study has some limitations. First, the sample size was small. Second, consistent use of in‐laboratory PSG device is necessary to ensure data consistency. Additionally, the study design was non‐randomized and without controls, which causes risks of selection bias and confounding factors contributing to the success rate of the procedure. We will continue to enroll patients with OSA and follow‐up with postoperative patients to obtain more robust conclusions. Moreover, a multicenter prospective study is required to obtain a high level of evidence.

## 5 Conclusion

In conclusion, the combination of modified UPPP and CCT can achieve an efficiency rate of 66.7% at a minimum 2 years postoperatively, demonstrating that the long‐term outcome of this procedure remains promising. However, a baseline AHI higher than 33.15 events/h predicts an unfavorable the long‐term outcome for UPPP plus CCT, and the procedure should be chosen with caution.

## Author Contributions

**Ren‐Hui Chen and Wei‐Qi Chen:** acquisition, analysis, interpretation of data. **Ren‐Hui Chen** and **Qian Cai:** drafting and editing the manuscript. **Xiao‐Ming Huang:** editing manuscript.

## Ethics Statement

The study was conducted in accordance with the Declaration of Helsinki and approved by the Ethics Committee of Sun Yat‐sen Memorial Hospital of Sun Yat‐sen University.

## Consent

Informed consent was obtained from all subjects involved in the study.

## Conflicts of Interest

Professor Xiao‐Ming Huang is a member of the *World Journal of Otorhinolaryngology – Head & Neck Surgery* (*WJOHNS*) editorial board and is not involved in the peer review process of this article.

## Data Availability

The data that support the findings of this study are available from the corresponding author upon reasonable request.
